# Effects of Heterogeneous Competitor Distribution and Ramet Aggregation on the Growth and Size Structure of a Clonal Plant

**DOI:** 10.1371/journal.pone.0068557

**Published:** 2013-07-02

**Authors:** Bi-Cheng Dong, Jiu-Zhong Wang, Rui-Hua Liu, Ming-Xiang Zhang, Fei-Hai Yu

**Affiliations:** 1 School of Nature Conservation, Beijing Forestry University, Beijing, China; 2 School of Forestry, Beijing Forestry University, Beijing, China; Centro de Investigación y de Estudios Avanzados, Mexico

## Abstract

Spatially heterogeneous distribution of interspecific competitors and intraspecific aggregation of offspring ramets may affect the growth and size structure of clonal plant populations, but these have been rarely studied. We conducted a greenhouse experiment in which we grew a population of eight offspring ramets (plants) of the stoloniferous clonal plant *Hydrocotyle vulgaris* aggregately or segregately in two homogeneous treatments with or without a competing grass *Festuca elata* and a heterogeneous treatment with a patchy distribution of the grass. In patchy grass treatments, *H. vulgaris* produced markedly more biomass, ramets and stolons in open patches (without grasses) than in grass patches, but displayed lower size variations as measured by coefficient of variation of biomass, ramets and stolons among the eight plants. In open areas, *H. vulgaris* produced statistically the same amounts of biomass and even more stolons and showed higher size variations in patchy grass treatments than in open (no grass) treatments. In grass areas, *H. vulgaris* grew much worse and displayed higher size variations in patchy grass treatments than in full grass treatments. Ramet aggregation decreased the growth of *H. vulgaris* in open treatments and in both open and grass patches in patchy grass treatments, but had little effect in full grass treatments. Ramet aggregation had little effect on size variations. Therefore, heterogeneous distribution of competitors can affect the growth and size structure of clonal plant populations, and ramet aggregation may decrease population growth when they grow in open environments or heterogeneous environments with a patchy distribution of interspecific competitors.

## Introduction

In natural habitats, clonal plants can produce horizontal structures (stolons, rhizomes or roots) to connect individual ramets, thereby experiencing spatially heterogeneous environments with microsites of different qualities [1]–[3]. Such environments are often created by an uneven distribution of both abiotic and biotic factors [4]–[7]. In heterogeneous environments, clonal plants may exploit high quality microsites via producing more ramets and concentrating more shoot or root biomass in such microsites, or avoid low quality microsites by veering away from them [2], [8]–[11]. Such responses may affect not only the growth and size of individual plants, but also the productivity and structure of clonal plant populations [6], [9], [12]–[15].

Plant populations may vary greatly in their responses to environmental heterogeneity [16]–[23]. It is commonly found that plant populations under soil nutrient heterogeneous conditions can gain greater biomass and individual size than those under homogenous conditions [18], [22]. On the other hand, Casper and Cahill (1998) found that soil nutrient heterogeneity hardly affected populations of *Abutilon theophrasti*, and Hagiwara *et al*. (2010) found that heterogeneity in water availability decreased biomass of *Perilla frutescens* populations. So far, however, few studies have tested the effect of spatial heterogeneity created by neighbor plants (e.g. patchy distribution of plants) on the growth and size structure of clonal plant populations.

One effect of neighboring plants is to create spatial heterogeneity in soil nutrients [6], [24]–[26], which is known to affect the growth of clonal plants [18], [27]. Neighboring plants can also form spatial heterogeneity in other features. They can act as physical obstacles because their root systems may block the spread of belowground rhizomes and tubers of coexisting clonal plants and their aboveground shoot systems may affect the distributions of the shoots [24], [28]. Neighboring plants may also interfere and suppress the normal root growth of co-occurring clonal plants by releasing nonspecific diffusible root exudates [29]–[31].

Ramet aggregation *vs.* segregation is another factor likely to affect the growth and size structure of clonal plant populations. Dispersal of clonal offspring ramets is usually limited compared to seed dispersal [32]. Thus, offspring ramets are often locally aggregated, and more likely close to intraspecific rather than interspecific neighbors [32], [33]. However, the effect of intraspecific aggregation of offspring ramets on plant population dynamics is commonly related to the competitive ability of neighbors [34], [35]. When interspecific competition is weaker than intraspecific competition or when interspecific competition is absent, plants that produce offspring ramets with short dispersal may be seriously inhibited by intraspecific aggregation. In contrast, when interspecific competition is stronger than intraspecific competition, offspring ramets may benefit from intraspecific aggregation because aggregation may prevent them from being directly interfered by potential aggressive interspecific neighbors. Therefore, we hypothesize that, when confronted with interspecific competitors, clonal plants grow better and show less size variation when offspring ramets are aggregated than when they are segregated. To our knowledge, however, few studies have explicitly tested the effect of intraspecific aggregation of offspring ramets on the growth and size structure of clonal plant populations [36], [37], especially in environments with a patchy distribution of competitors.

We conducted a greenhouse experiment in which we grew a population of eight offspring ramets of the stoloniferous clonal plant *Hydrocotyle vulgaris* aggregately or segregately in two homogeneous environments with or without competing neighbors *Festuca elata* as well as in a heterogeneous environment with a patchy distribution of *F. elata*. We specifically asked: (1) Does heterogeneous distribution of interspecific competitors affect the growth and size structure of *H. vulgaris*? (2) Does intraspecific aggregation of offspring ramets affect the growth and size structure of *H. vulgaris*?

## Materials and Methods

### The Species

*Hydrocotyle vulgaris* L. (Araliaceae) is a perennial clonal herb and commonly occurs in bogs, valleys and dune grassland [38]. It can produce plagiotropic stems (i.e., stolons). Each node along the stolons has the potential to form a ramet that consists of a leaf and adventitious roots. The dispersal of *H. vulgaris* relies mainly on vegetative means rather than sexual reproduction [39]. In the field, *H. vulgaris* can produce extensive shoot systems and experience heterogeneous micro-environments created by either resource availability or aggregated distribution of neighboring plants [38], [40], [41]. *H. vulgaris* plants used in this experiment were collected from a wetland in the suburbs of Hangzhou, Zhejiang Province, China, and propagated vegetatively in a greenhouse at Forest Science Co. Ltd. of Beijing Forestry University. The sampling site did not belong to the part of any farms or national parks, so we did not need any relevant permissions/permits for collecting plant samples.

### The Experiment

**Figure 1:**
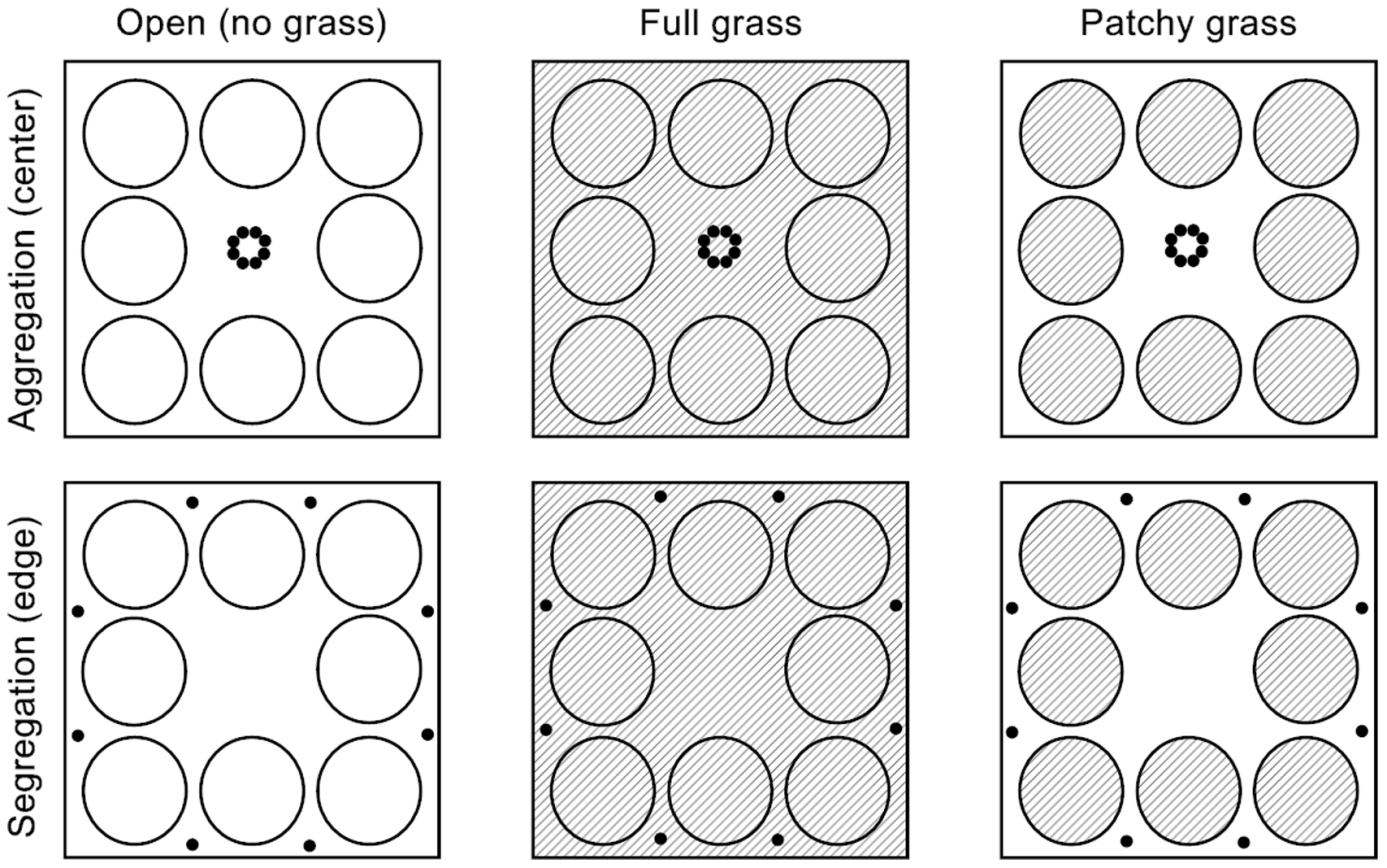
Schematic representation of the experimental design. The experiment had three neighbor interference treatments [open - there was no grass in the square container, full grass - the grass *Festuca elata* (hatched) was grown in the entire container, and patchy grass - *F. elata* was grown in eight regularly spaced circular patches within the container] crossed with two ramet aggregation treatments [eight offspring ramets (black dots) of *Hydrocotyle vulgaris* were initially planted in aggregation (in the center of a container) or in segregation (closer to the edges)].

The experiment took a factorial design and had two factors: neighbor inference and ramet aggregation. There were three levels of neighbor interference (open - there was no grass in the square container, full grass - the grass *Festuca elata* was grown in the entire container, and patchy grass - *F. elata* was grown in eight regularly spaced circular patches within the container) and two levels of ramet aggregation (eight offspring ramets of *H. vulgaris* were initially planted in aggregation and were close to the center of the container, or in segregation and were closer to the inner borders of the container; Fig. 1). Each treatment had six replicate containers (40 cm long×40 cm wide×60 cm high) and thus there were 36 plastic containers. In each container, eight 0.2-cm-thick PVC tubes (10.5 cm in inner diameter×13 cm in height) were installed at regular, fixed positions, covering 43% surface area of the container (Fig. 1). Each container was filled to a depth of 15 cm with a 1∶1 (v:v) mixture of sand and peat-based substrate (Pindstrup Seeding; Pindstrup Mosebrug A/S, Denmark), plus 1 g L^−1^ slow-release fertilizer (15N-11P-13K-2Mg; Osmocote 301, Scotts, USA). The PVC tubes were installed in the way that their tops were 2 cm below the soil surface so that the tubes could not hamper the horizontal spread of stolons and ramets of *H. vulgaris*. The installation of the tubes ensured that roots of the grasses in the grass patches (tubes) could not spread into the open areas in the patchy grass treatment. The 60 cm high containers guaranteed that stolons and ramets of *H. vulgaris* could not grow out of the containers.

On 16 April 2012, in 12 containers the whole area were evenly sown with *F. elata* seeds at a density of 30 g cm^−2^ (full grass), in another 12 containers only the area in the eight PVC tubers were sown with *F. elata* seeds (patchy grass), and in the remaining 12 containers no area was sown with *F. elata* seeds (open). After 10 days, *F. elata* seedlings reached a height of about 10 cm. On 27 April, 288 ramets of *H. vulgaris* were selected. The initial petiole length was 8.87±0.36 (mean ± SE, n = 18) cm and dry mass was 30.61±3.34 mg. The ramets were divided into six groups according to petiole length and plant size, with 48 ramets in each group. In each group the 48 ramets were randomly assigned to the six treatments and each replicate container had eight ramets. In one ramet aggregation treatment, the eight ramets were regularly planted in the places close to the center of the container within a circular area of 4 cm in diameter, and in the other ramet aggregation treatment they were planted in eight positions 2 cm away from the container borders (Fig. 1).

The experiment was conducted from 27 April to 7 July 2012. The mean temperature and relative humidity (mean ± SE) during the experiment were 26.21±0.33°C and 59.02±1.46% (iButton DS1923, Maxim Integrated Products, USA). During the experiment, sufficient tap water was added to each container to keep the soil moist.

### Measurements

At harvest, the new ramets produced by each initial plant (ramet) were interconnected by aboveground stolons, so we could harvest and measure the growth variables of the plants in each type of patches separately. For the homogeneous treatments (the open and full grass treatments), we counted number of ramets and number of stolons, measured total stolon length and weighed dry leaf, petiole, stolon and root mass of each *H. vulgaris* plant in each container. For the patchy grass treatments, each plant in a container was divided into three parts (initial plants that grew in the borders of the open and grass patches, offspring ramets that grew within the open patches, and offspring ramets that grew in the grass patches) and measured separately. All plant parts were oven-dried at 70°C for 72 h before weighing. Aboveground biomass (dry weight) of *F. elata* in the full grass treatments and the patchy grass treatments were also measured.

### Data Analysis

We calculated total biomass per unit area, number of ramets per unit area, stolon length per unit area and number of stolons per unit area in each container for all treatments. For the patchy grass treatments, we also calculated such growth variables for the grass patches and open patches separately. To measure size variations, we calculated CV (i.e., standard deviation of the eight clones divided by the mean values of the eight clones) in each container for all treatments based on total biomass, number of ramets, stolon length and number of stolons of the eight plants that originated from the eight initial ramets of *H. vulgaris* [42]. For the patchy grass treatments, we calculated CV of *H. vulgaris* in the grass patches as standard deviation of the eight plants divided by the mean values of the eight plants. Similarly, we also calculated CV of *H. vulgari*s in the open patches as standard deviation of the eight plants in the open patches divided by the mean values of the eight plants in the open patches.

First, we used two-way ANOVA to test the effects of neighbor interference (open, full grass and patchy grass) and status of ramet aggregation (aggregation and segregation) on the measures of growth (biomass, number of ramets, stolon length, number of stolons) and size variation (CV of biomass, CV of ramet number, CV of stolon length and CV of stolon number) of *H. vulgaris*. Second, we used two-way ANOVA to test the effects of patchy distribution of grasses and status of ramet aggregation on all measures of *H. vulgaris* grown in the grass patches/areas, and in these analyses we included variables of the plants in the full grass treatment and the corresponding variables of the plant parts in the grass patches in the patchy grass treatment. Similarly, we used two-way ANOVA to test the effects of patchy distribution of grasses and status of ramet aggregation on all measures of *H. vulgaris* grown in the open patches/areas, and in these analyses we included variables of the plants in the open treatment and the corresponding variables of the plant parts in the open patches in the patchy grass treatment. Third, we employed repeated-measures ANOVA to test the effects of patch type (open patches and grass patches) and status of ramet aggregation on all measures, and in these analyses we only included the patchy grass treatments [43]. Patch type was treated as a repeated variable because the open and grass patches in each container were not independent from each other. By accident, we lost one replicate in the homogeneous grass treatment with the *H. vulgaris* ramets were initially planted segregately.

We also used two-way ANOVA to test the effects of the distribution type of *F. elata* (full grass and patchy grass) and ramet aggregation of *H. vulgaris* (aggregation and segregation) on aboveground biomass of *F. elata*. We further used Principal Component Analysis (PCA) to assess whether the eight response variables (biomass, number of ramets, stolon length, number of stolons, CV of biomass, CV of ramet number, CV of stolon length and CV of stolon number) of *H. vulgaris* were grouped by the correlations between the response variables either at the whole container level or at the patch level [44].

All analyses were conducted using SPSS 16.0 (SPSS, Chicago, IL, USA). The effects were considered significant if *P*<0.05.

## Results

### Effects of Neighbor Interference and Ramet Aggregation at the Whole Container Level

**Figure 2:**
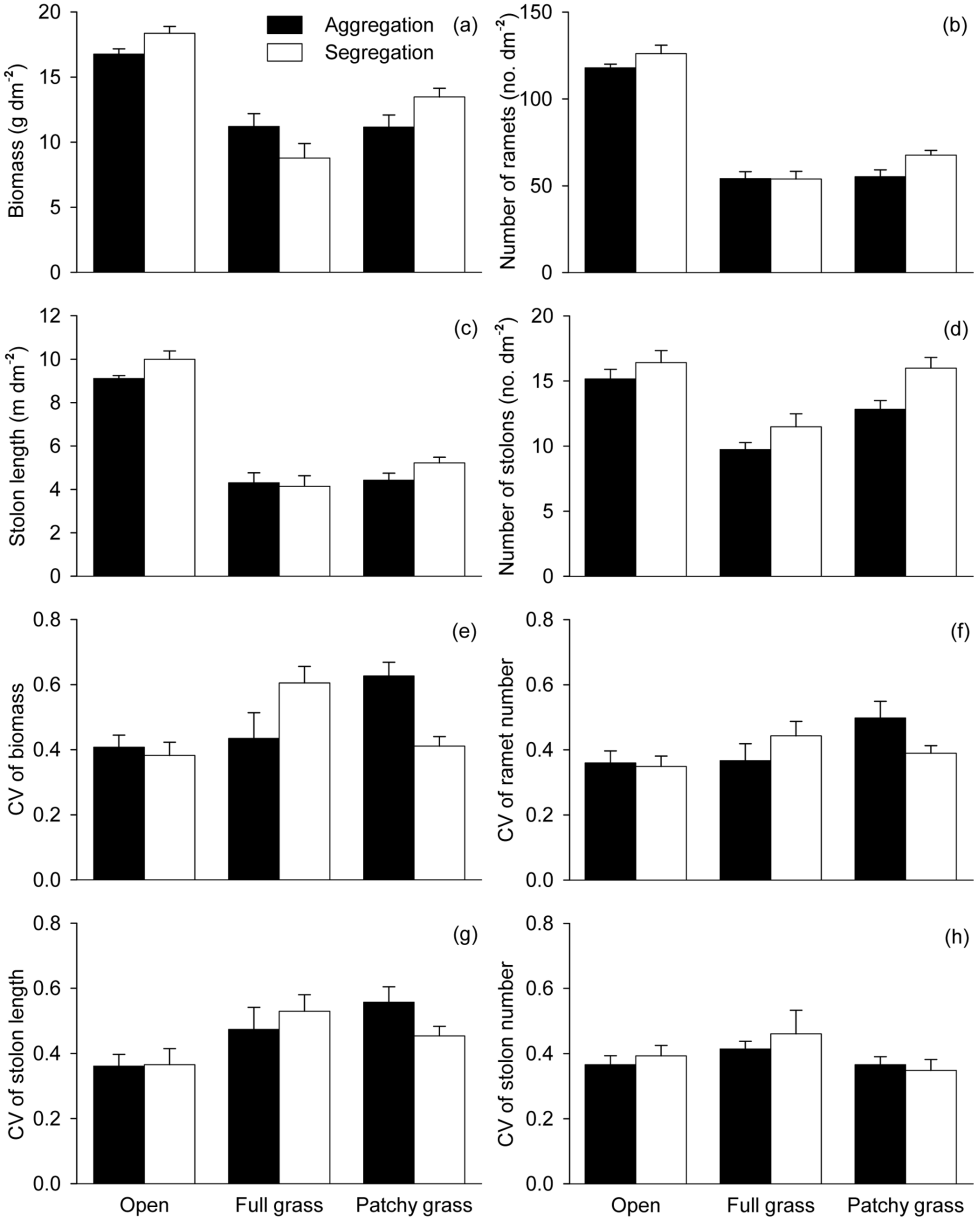
Effects of neighbor interference (open *vs.* full grass *vs.* patchy grass) and ramet aggregation (aggregation *vs.* segregation) on the growth (a–d) and size inequality (e–h) of the *Hydrocotyle vulgaris* populations. Error bars show +1 SE.

All growth measures (biomass, number of ramets, stolon length and number of stolons) of *H. vulgaris* were the largest in the open treatments, smallest in the full grass treatments, and intermediate in the patchy grass treatments (Table 1, Fig. 2a–d). *H. vulgaris* produced more ramets and stolons and tended to (*P*<0.1) produce longer stolons when the ramets were grown initially in segregation than when they were grown in aggregation (Table 1, Fig. 2b–d). *H. vulgaris* produced more biomass when offspring ramets were segregated than aggregated in the open and patchy grass treatments, but less in the full grass treatments (Table 1, Fig. 2a).

**Table 1:** ANOVAs results for effects neighbor interference (open *vs.* full grass *vs.* patchy grass) and status of ramet aggregation (aggregation *vs.* segregation) on the growth and size variation of *Hydrocotyle vulgaris* at the whole container level.

	Neighbor (N)		Aggregation (A)		N×A	
	*F*_2,29_	*P*	*F*_1,29_	*P*	*F*_2,29_	*P*
Biomass	47.94	<0.001	0.59	0.448	5.08	0.013
Number of ramets	198.52	<0.001	4.89	0.035	1.44	0.254
Stolon length	140.05	<0.001	3.15	0.087	1.35	0.276
Number of stolons	22.95	<0.001	10.44	0.003	0.84	0.443
CV of biomass	4.32	0.023	0.35	0.560	7.56	0.002
CV of ramet number	2.42	0.107	0.17	0.680	2.44	0.105
CV of stolon length	5.71	0.008	0.14	0.713	1.41	0.261
CV of stolon number	2.52	0.098	0.39	0.536	0.40	0.672

CV of biomass and stolon length were significantly higher in the full grass and the patchy grass treatments than in the open treatments, but did not differ between the full grass and patchy grass treatments (Table 1, Fig. 2e, g). CV of ramet number or stolon number was not affected by neighbor interference (Table 1, Fig. 2f, h). However, ramet aggregation affected none of the four size variation measures, except that there was a significant interaction effect on CV of biomass (Table 1, Fig. 2e–h).

### Effects of Heterogeneity and Ramet Aggregation in Open Patches/areas

**Figure 3:**
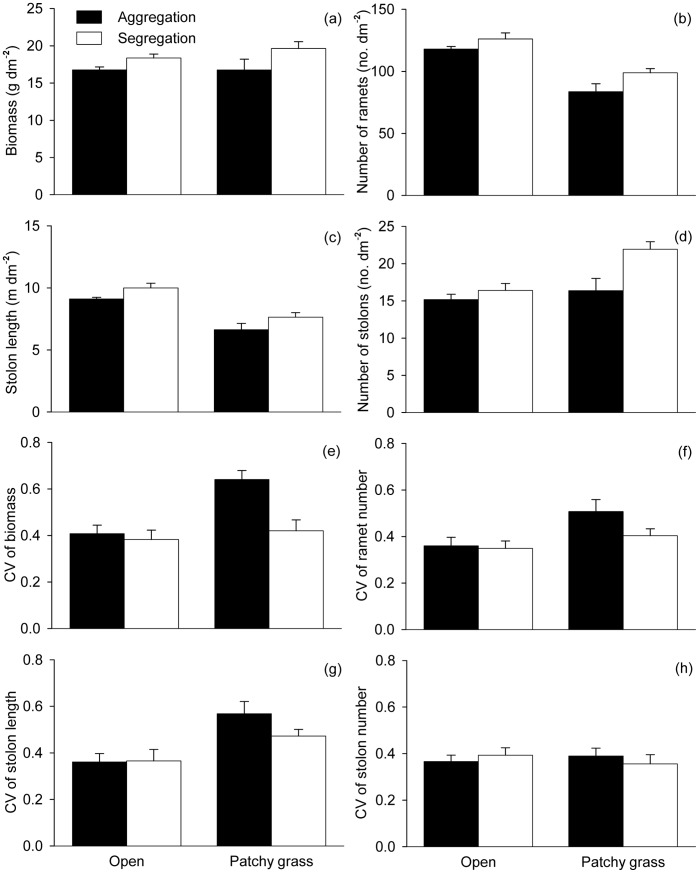
Effects of spatial heterogeneity (open *vs.* patchy grass) and ramet aggregation (aggregation *vs.* segregation) on the growth (a–d) and size variation (e–h) of the *Hydrocotyle vulgaris* populations grown in the open areas. Error bars show +1 SE.

In the open areas, *H. vulgaris* produced more stolons in the patchy grass than in the open treatments (Table 2, Fig. 3d). However, compared with the open treatments, *H. vulgaris* produced statistically the same amounts of biomass and fewer ramets and shorter stolons in the patchy grass treatments (Table 2, Fig. 3a–c). All growth measures were greater when the ramets were grown segregately than aggregately (Table 2, Fig. 3a–d).

**Table 2:** ANOVAs results for effects of spatial heterogeneity (open *vs.* patchy grass) and status of ramet aggregation (aggregation *vs.* segregation) on the growth and size variation of *Hydrocotyle vulgaris* in the open patches/areas.

	Heterogeneity (H)		Aggregation (A)		H×A	
	*F*_1,20_	*P*	*F*_1,20_	*P*	*F*_1,20_	*P*
Biomass	0.47	0.500	5.94	0.024	0.49	0.492
Number of ramets	47.00	<0.001	6.81	0.017	0.60	0.448
Stolon length	41.97	<0.001	6.49	0.019	0.03	0.874
Number of stolons	8.84	0.008	9.06	0.007	3.63	0.071
CV of biomass	10.92	0.004	8.96	0.007	5.64	0.028
CV of ramet number	6.90	0.016	2.20	0.153	1.43	0.246
CV of stolon length	13.48	0.002	1.14	0.299	1.35	0.258
CV of stolon number	0.04	0.848	0.01	0.916	0.83	0.372

In the open areas, *H. vulgaris* displayed higher CV of biomass, ramet number and stolon length in the patchy grass than in the open treatments (Table 2, Fig. 3e–g), and for CV of biomass such an effect of heterogeneity was larger when initial ramets were aggregated than segregated (Table 2, Fig. 3e). Ramet aggregation affected none of the size variation measures except CV of biomass (Table 2, Fig. 3e–h).

### Effects of Heterogeneity and Ramet Aggregation in Grass Patches/areas

**Figure 4:**
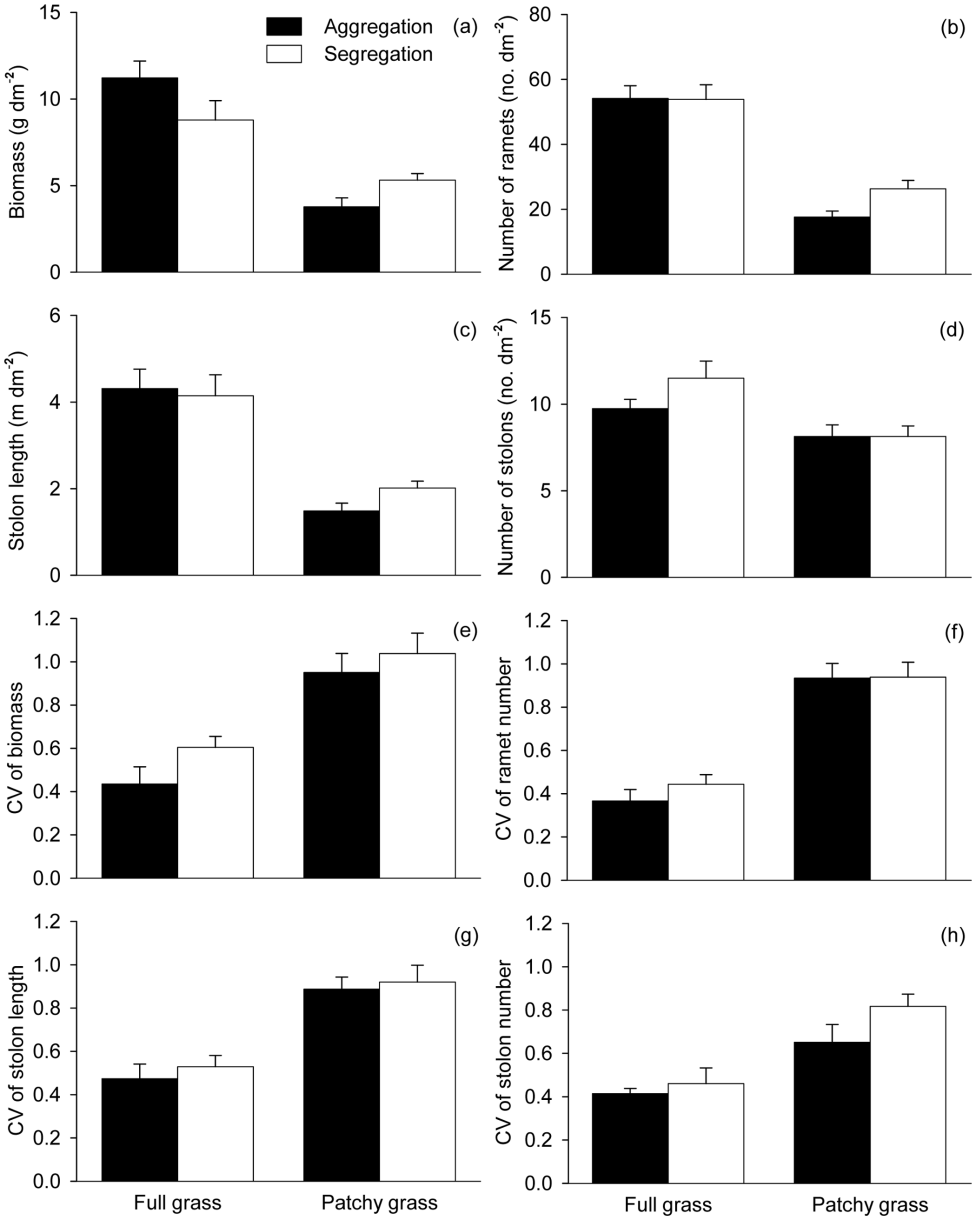
Effects of spatial heterogeneity (full grass vs. patchy grass) and ramet aggregation (aggregation *vs.* segregation) on the growth (a–d) and size variation (e–h) of the *Hydrocotyle vulgaris* populations grown in the grass areas. Error bars show +1 SE.

In the grass areas, all growth measures were greater in the full grass than in the patchy grass treatments (Table 3, Fig. 4a–d), and for biomass such an effect of spatial heterogeneity was larger when the ramets were initially in aggregation than in segregation (Table 3, Fig. 4a). Ramet aggregation did not affect number of ramets, stolon length or number of stolons (Table 3, Fig. 4b–d).

**Table 3:** ANOVAs results for effects of spatial heterogeneity (full grass *vs.* patchy grass) and status of ramet aggregation (aggregation *vs.* segregation) on the growth and size variation of *Hydrocotyle vulgaris* in the grass patches/areas.

	Heterogeneity (H)		Aggregation (A)		H×A	
	*F*_1,19_	*P*	*F*_1,19_	*P*	*F*_1,19_	*P*
Biomass	49.10	<0.001	0.32	0.577	6.50	0.020
Number of ramets	95.68	<0.001	1.67	0.212	1.88	0.186
Stolon length	53.37	<0.001	0.28	0.601	1.04	0.321
Number of stolons	12.85	0.002	1.59	0.222	1.59	0.222
CV of biomass	33.10	<0.001	2.42	0.136	0.25	0.620
CV of ramet number	76.41	<0.001	0.45	0.508	0.35	0.564
CV of stolon length	37.64	<0.001	0.45	0.510	0.03	0.861
CV of stolon number	22.65	<0.001	2.91	0.104	0.92	0.351

In the grass areas, all measures of size variation were lower in the full grass than in the patchy grass treatments, but none was affected by ramet aggregation (Table 3, Fig. 4e–h).

### Effects of Patch Type and Ramet Aggregation in the Patchy Grass Treatments

**Figure 5:**
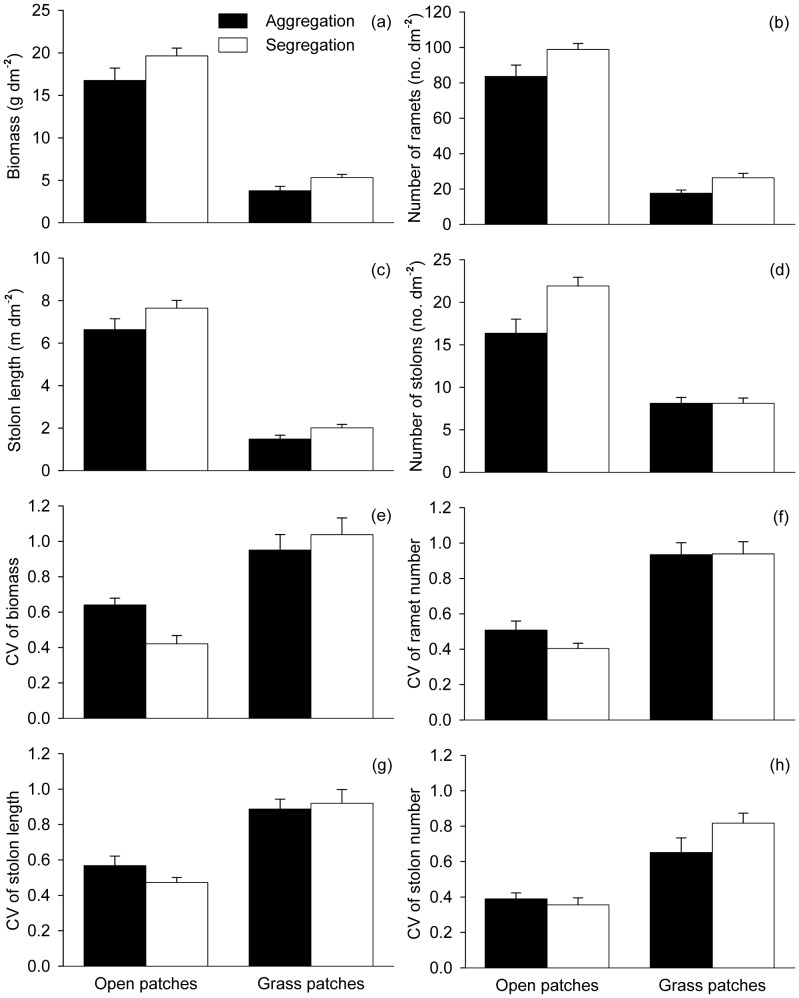
Effects of patch type (open patches *vs.* grass patches) and ramet aggregation (aggregation *vs.* segregation) on the growth (a–d) and size variation (e–h) of the *Hydrocotyle vulgaris* populations. Error bars show +1 SE.

In the patchy grass treatments, all growth measures were greater in the open patches than in the grass patches (Table 4, Fig. 5a–d). Compared with aggregation, initial ramet segregation significantly increased number of ramets and stolons and tended to (*P*<0.1) increase biomass and stolon length (Table 4, Fig. 5a–d).

**Table 4:** Repeated measure ANOVA results for effects of patch type (open patches *vs.* grass patches) and status of ramet aggregation (aggregation *vs.* segregation) on the growth and size variation of *Hydrocotyle vulgaris.*

	Patch type (T)		Aggregation (A)		T×A	
	*F*_1,10_	*P*	*F*_1,10_	*P*	*F*_1,10_	*P*
Biomass	310.74	<0.001	4.53	0.059	0.76	0.403
Number of ramets	403.25	<0.001	7.34	0.022	0.88	0.369
Stolon length	365.20	<0.001	4.04	0.072	0.73	0.413
Number of stolons	86.21	<0.001	8.94	0.014	5.49	0.041
CV of biomass	38.52	<0.001	0.97	0.349	4.21	0.067
CV of ramet number	58.67	<0.001	0.98	0.346	0.75	0.406
CV of stolon length	36.78	<0.001	1.55	0.242	2.82	0.124
CV of stolon number	49.30	<0.001	0.29	0.602	1.37	0.269

All measures of size variation were lower in the open patches than in the grass patches, but none was affected by ramet aggregation (Table 4, Fig. 5e–h).

### The Response Pattern of H. vulgaris to Patch Type and Ramet Aggregation

**Figure 6:**
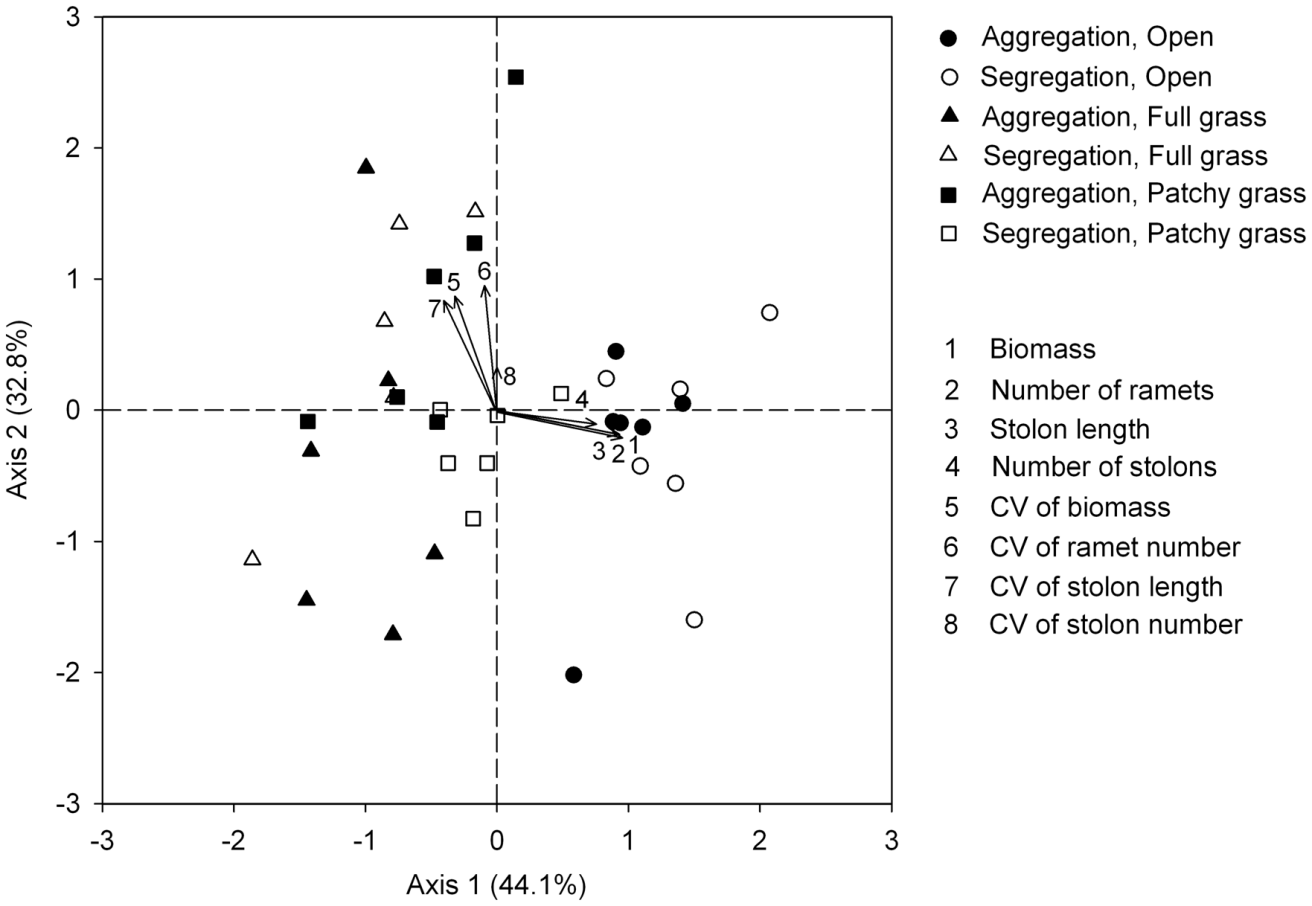
Scatter plot from Principal Component Analysis of the *Hydrocotyle vulgaris* populations at the whole container level. Axis 1 and 2 explained 44.1% and 32.8% of the total variance, respectively. Response variable scores (arrows) are given.

At the whole container level, the first PCA axis was identified as an axis of the growth of the population, accounting for 44.1% of the total variance, and PCA axis 2 was identified as an axis of the size structure of the population, accounting for 32.8% of the total variance. The open treatments were located at the higher end of PCA axis 1 and clearly separated from the other treatments (Fig. 6).

**Figure 7:**
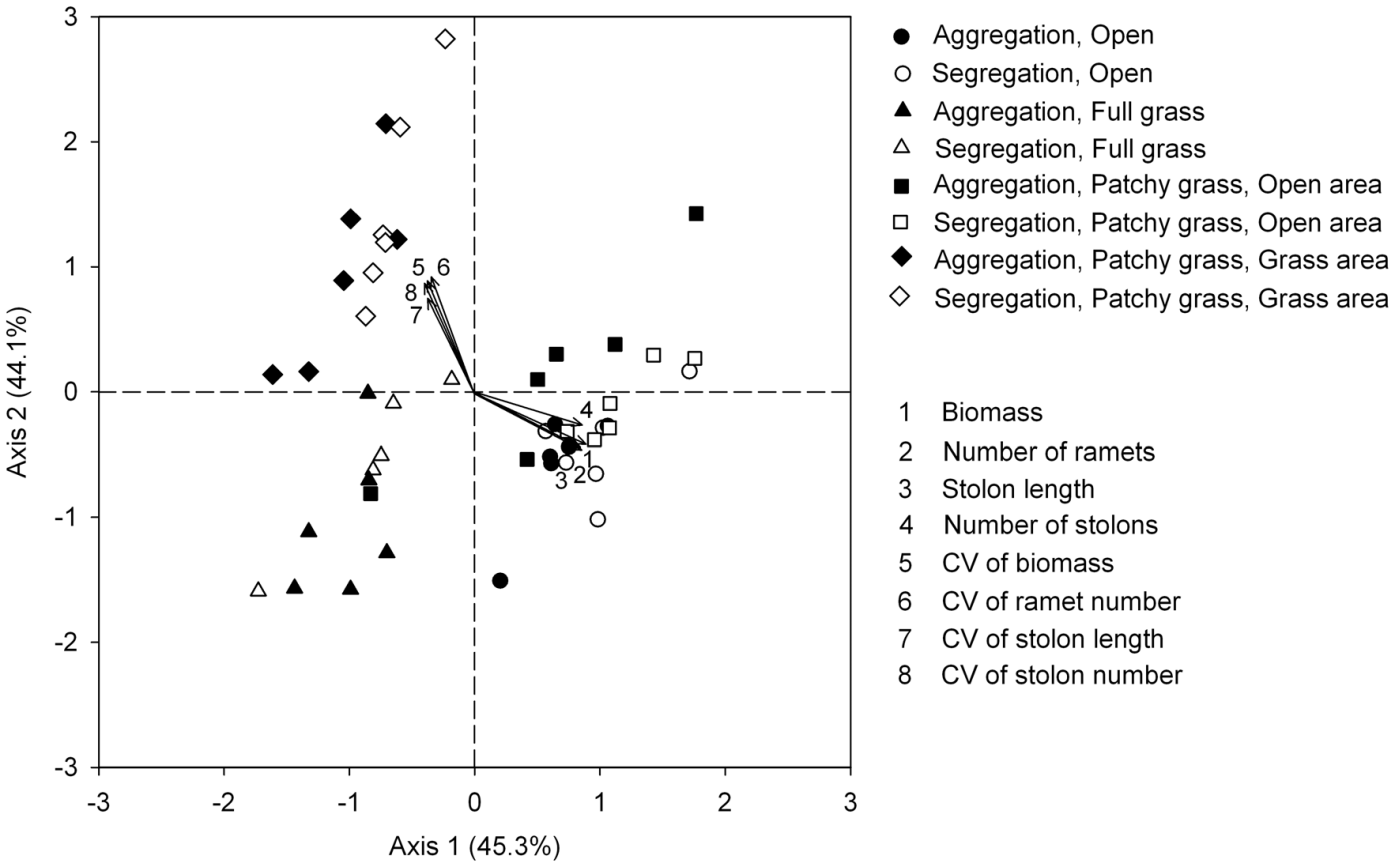
Scatter plot from Principal Component Analysis of the *Hydrocotyle vulgaris* populations at the patch level. Axis 1 and 2 explained 45.3% and 44.1% of the total variance, respectively. Response variable scores (arrows) are given.

At the patch level, PCA axis 1 explained 45.3% of the total variation and was related with the growth of the population, and PCA axis 2 explained 44.1% of the total variation and was related with the size structure of the population (Fig. 7). The open treatments and the corresponding open areas in the grass patchy treatments were located at the higher end of PCA axis 1, while the full grass treatments and the corresponding grass areas in the grass patchy treatments were located at the lower end of PCA axis 1. The open areas in the grass patchy treatments were located at the higher end of PCA axis 2, and the full grass treatments were at the lower end of PCA axis 2. These results generally agreed with results from univariate ANOVAs.

### The Growth of F. elata

Aboveground biomass of *F. elata* was significantly affected by distribution type (*F*_1,19_ = 322.22; *P*<0.001), but not ramet aggregation of *H. vulgaris* (*F*_1,19_<0.01; *P* = 0.969) or the interaction effect of ramet aggregation by distribution type (*F*_1,19_ = 0.13; *P* = 0.721). Aboveground biomass of *F. elata* in the patchy grass treatments (5.96±0.18 g dm^−2^; mean ± SE) was significantly higher than in the full grass treatments (1.16±0.19 g dm^−2^; mean ± SE).

## Discussion

The growth of the *H. vulgaris* plants was severely inhibited when they were grown with the grass *F. elata* (Fig. 6), suggesting that the presence of *F. elata* caused intense interspecific competition likely for light [45] and physical space [28] and possibly also for soil nutrients [24]. Moreover, the presence of interspecific competitors increased size variations as indicated by increased CV of biomass and stolon length in the full grass and patchy grass treatments as compared with those in the open treatments (Table 1, Fig. 2e, g). These results also mean that the individual plants of the *H. vulgaris* populations were suppressed unequally by interspecific interference of *F. elata*, possibly due to the difference in the time to produce offspring ramets between individuals. Plants that produced offspring ramets earlier could act promptly (e.g., enhancing petiole length to increase light acquisition at the early development) and thus capture more light before the interspecific suppression became serious, while plants that produced offspring ramets later would be shaded and suppressed by either *F. elata* or intraspecific neighbors [40]. Thus, interspecific competition also altered population structure of *H. vulgaris* [46], [47].

### Effects of Heterogeneous Distribution of Competitors

In environments with a patchy distribution of grasses, *H. vulgaris* grew much worse in the grass patches than in the open patches (Table 4, Figs. 5 and 7). Moreover, *H. vulgaris* produced much less biomass and fewer ramets in the grass patches in the patchy grass treatments than in the full grass treatments (Table 3, Fig. 4). These results suggest that, when grown in environments with a patchy distribution of grasses, *H. vulgaris* reduced the chance to grow into the grass patches. Such a response may be a passive process, because the aggregation of *F. elata* in the patchy grass treatments could produce more aboveground biomass than in the full grass treatments. Thus, the suppression of *H. vulgaris* by *F. elata* was more severe in the patchy grass treatments than in the full grass treatments. The results also indicate that connections to ramets in the open patches could not contribute much to the growth and clonal reproduction of the ramets in the grass patches [48], [49].

On the other hand, the patchy grass environments might also have triggered active foraging responses of *H. vulgaris* to minimize the interspecific competition [24], [28], [49]. This argument is supported further by the fact that in the patchy grass treatment *H. vulgaris* markedly decreased stolon branching in the grass patches, and tended to expand their stolons along the edge of grass patches rather than across the patches (personal observations). Similar foraging responses were reported in at least three previous studies [24], [28], [50], and these active foraging responses are thought to be adaptive for clonal plants to improve the space-use efficiency in heterogeneous environments with a patchy distribution of neighbor plants [51].

*H. vulgaris* produced statistically the same amounts of biomass and even increased stolon production in the open areas in the patchy grass treatments than in the open treatments. These results suggest that connections to ramets in the grass patches could not cause significant costs to the growth of the ramets in the open patches [48], [52], [53]. The likely reason is that resource transportation to the ramets in the grass patches was rather low or even did not exist [48], [49], as shown also by the fact that connections to the ramets in the open patches did not increase the growth of the ramets growing in the grass patches.

In environments with a patchy distribution of grasses, *H. vulgaris* showed markedly higher size variations in the grass patches than in the open patches (Figs. 5e–h and 7). Moreover, *H. vulgaris* had higher size variations in both open and grass patches in the patchy grass treatment than in the corresponding areas in the open and full grass treatments (Tables 2 and 3, Figs. 3e–g and 4e–h). These results provide the first evidence that spatial heterogeneity in distribution of interspecific competitors can markedly alter size structure of clonal plant populations. One obvious consequence of spatial heterogeneity in competitor distribution is to cause variations in the difficulty of resource acquisition in different types of patches [9], [22], [54]. Physical space is also considered as a resource for plants [55]–[57]. Therefore, another possible consequence is that spatial heterogeneity in competitor distribution generates variations in the availability of physical space and thus variations in the difficulty for plants to find available space [55]–[57]. Such variations further resulted in the changes in the size structure of the clonal plant populations.

### Effects of Aggregation of Offspring Ramets

Compared with aggregation, initial ramet segregation significantly increased biomass, ramet and stolon production of *H. vulgaris* in the open treatments (Tables 1, Fig. 2a–d), suggesting that ramet aggregation negatively affects the growth of clonal plant populations when they grow in an open environment or spread into open patches. Similarly, Lenssen *et al*. (2005) found that ramet aggregation reduced the competitive ability of *Agrostis stolonifera*. The likely reason is that, when interspecific competition was relative weak or absent, aggregation of offspring ramets aggravated either self-competition between different ramets within the same clone or intraspecific competition between different clones by increasing the overlapping zones of influence [58], [59].

Ramet aggregation had little effect on the growth of *H. vulgaris* in the full grass treatments (Table 1, Fig. 2a–d), suggesting that ramet aggregation cannot benefit clonal plants when they grow with a homogeneous distribution of interspecific neighbors [34], [35]. These results seem not to support the view that intraspecific aggregation can change the influence frequency of inter- *vs.* intraspecific encounters and thus contribute to species coexistence [36], [60]–[62]. However, because there is only one seed-sowing density treatment of *F. elata* in the experiment, we are not sure whether the benefit of intraspecific aggregation is density dependent and whether it will become more important when the seed-sowing density is lower [34], [35]. Further studies that compare the effect of intraspecific aggregation under different seed-sowing density treatments may help us understand the potential mechanism.

Ramet aggregation in the patchy grass treatments had even negative effects on the growth of *H. vulgaris* at the patch and the container levels (Tables 1–4, Figs. 2a–d, 3a–d, 4a–d and 5a–d). When intraspecific competitor *F. elata* was restricted in fixed regions in the patchy grass treatments, *H. vulgaris* could easily occupy the open areas and maintain the long-term control of local resources by producing new ramets, thereby resulting in the strong spatial segregation between different species [34], [35]. Under such circumstances, the effect of ramet aggregation in the open areas seems to determine the performance of *H. vulgaris* in the grass area and even at the whole container level.

Therefore, spatial distribution of offspring ramets is an important factor to determine the establishment of clonal plant populations when they are introduced in an open environment or an environment with a patchy distribution of interspecific competitors, but may not be so when they invade into a closed community with a homogeneous distribution of competitors. However, we found that ramet aggregation had little effect on size variations (Tables 2–>4, Figs. 3f–h, 4e–h and 5e–h). Thus, there is no evidence that ramet aggregation can affect size structure of clonal plant populations.

### Conclusions

Both spatially heterogeneous distribution of interspecific competitors and intraspecific aggregation of offspring ramets can greatly affect the growth of clonal plant populations, and heterogeneous distribution of competitors can also alter population size structure. However, due to the limited area of the experimental container, we cannot completely rule out the potential edge effect caused by initial ramet positions (e.g., the ramets planted segregately were located also more closely to the edges of the containers). In further studies, therefore, larger containers should be used so that an extra buffer can be added to reduce the potential confounding effects of edges [36], [47].
